# Functional Upregulation of Nav1.8 Sodium Channels on the Membrane of Dorsal Root Ganglia Neurons Contributes to the Development of Cancer-Induced Bone Pain

**DOI:** 10.1371/journal.pone.0114623

**Published:** 2014-12-11

**Authors:** Xiao-Dan Liu, Jing-Jing Yang, Dong Fang, Jie Cai, You Wan, Guo-Gang Xing

**Affiliations:** 1 Neuroscience Research Institute, Peking University, Beijing, People's Republic of China; 2 Department of Neurobiology, School of Basic Medical Sciences, Peking University Health Science Center, Beijing, People's Republic of China; 3 Key Laboratory for Neuroscience, Ministry of Education and Ministry of Health, Beijing, People's Republic of China; University of Pittsburgh School of Medicine, United States of America

## Abstract

We have previously reported that enhanced excitability of dorsal root ganglia (DRG) neurons contributes to the development of bone cancer pain, which severely decreases the quality of life of cancer patients. Nav1.8, a tetrodotoxin-resistant (TTX-R) sodium channel, contributes most of the sodium current underlying the action potential upstroke and accounts for most of the current in later spikes in a train. We speculate that the Nav1.8 sodium channel is a potential candidate responsible for the enhanced excitability of DRG neurons in rats with bone cancer pain. Here, using electrophysiology, Western blot and behavior assays, we documented that the current density of TTX-R sodium channels, especially the Nav1.8 channel, increased significantly in DRG neurons of rats with cancer-induced bone pain. This increase may be due to an increased expression of Nav1.8 on the membrane of DRG neurons. Accordantly, blockade of Nav1.8 sodium channels by its selective blocker A-803467 significantly alleviated the cancer-induced mechanical allodynia and thermal hyperalgesia in rats. Taken together, these results suggest that functional upregulation of Nav1.8 channels on the membrane of DRG neurons contributes to the development of cancer-induced bone pain.

## Introduction

Bone cancer pain resulting from primary tumors or tumors that metastasize to bones is one of the most severe and intractable types of cancer pain, which decreases the quality of life of patients [1]. The mechanisms underlying the development of bone cancer pain remain largely unknown.

Recently, we and others have found that thermal hyperalgesia and mechanical hypersensitivity in murine models of bone cancer pain are associated with enhanced excitability of primary nociceptive DRG neurons [2], [3]. The responses of nociceptors to noxious stimuli are encoded by action potentials whose genesis and propagation are dependent on voltage-gated sodium channels. Thus, aberrant expression patterns of these channels and inherited sodium channelopathies have been linked to neuropathic and inflammatory pain [4]. Adult DRG neurons can express both tetrodotoxin-sensitive (TTX-S) and tetrodotoxin-resistant (TTX-R) sodium channels. Among the latter, the TTX-R sodium channel Nav1.8 is specifically expressed on sensory neurons [5], [6]. Hence, Nav1.8 is one of the most attractive targets for the development of new pharmaceutical agents to treat pain.

Nav1.8 produces a slow-inactivating, rapid-repriming TTX-R sodium current with depolarized activation and inactivation voltage-dependency [6], [7]. Nav1.8 contributes most of the sodium current underlying the action potential upstroke in neurons that expresses the channel [8], [9]. The biophysical properties of Nav1.8, its critical role in repetitive firing, and its presence in free nerve endings, where pain signaling is initiated, suggest that Nav1.8 can significantly influence nociceptors excitability, thus contributing to pain. The role of Nav1.8 in neuropathic and inflammatory pain is well reviewed by Dib-Hajj et al. [4]. However, whether Nav1.8 contributes to the development of cancer-induced bone pain is largely unknown. Recently, Qiu and colleagues [10] have observed an increased expression of Nav1.8 within DRG in a rat model of Walker 256 tumor cell-induced bone cancer pain, suggesting the potential involvement of Nav1.8 in the development of cancer-induced bone pain. In this study, using electrophysiology, Western blot and pharmacological behavior methods, we provide evidence showing that functional upregulation of Nav1.8 channels on the membrane of DRG neurons contributes to the development of cancer-induced bone pain.

## Materials and Methods

### Animals

Adult female Sprague-Dawley rats weighing 180–220 g at the beginning of the experiments were provided by the Department of Experimental Animal Sciences, Peking University Health Science Center. The rats were housed in separated cages with free access to food and water. The room temperature was kept at 24±1°C under natural light/dark cycle. All experimental animal procedures were conducted in accordance with the guidelines of the International Association for the Study of Pain [11] and were approved by the Animal Care and Use Committee of Peking University.

### Inoculation of tumor cells

MRMT-1 rat mammary gland carcinoma cells were cultured in medium containing RPMI 1640 (Hyclone, USA) and 10% foetal bovine serum. Cells were released from the plastic by brief exposure to 0.25% (weight/volume) trypsin (Gibco, USA), and then prepared for injection as follows: the cells were firstly collected by centrifugation of 10 ml of medium for 3 min at 1000 rpm. The resulting pellet was then resuspended in 1 ml of phosphate-buffered saline (PBS) and cells were counted using a haemocytometer. Next, cells were diluted to achieve the final concentration for injection and kept on ice until injected into animals. A rat model of bone cancer pain was established by intratibial injection of syngeneic MRMT-1 cells as previously described [12]. Briefly, after anesthetized with chloral hydrate (0.3 g/kg, i.p.), the rat left tibia was carefully exposed, and a 23-gauge needle was inserted into the intramedullary canal of the bone. It was then removed and replaced with a long thin blunt needle attached to a 10-µl Hamilton syringe containing the medium to be injected. A volume of 4 µl MRMT-1 rat mammary gland carcinoma cells (4×10^4^) or vehicle (PBS) was injected into the tibial bone cavity. Following injection the site was sealed with bone wax, and the wound was finally closed. None of the animals showed signs of motor dysfunction after implantation of tumor cells.

### Whole-cell patch clamp recording

Neurons were isolated from L4 and L5 DRG of adult rats using methods as described in our previous studies [3], [13]. Briefly, freshly dissected ganglia were minced and washed in cold, oxygenated Dulbecco Modified Eagle Medium (DMEM; Sigma), and were then subjected to collagenase (3 mg/ml, type IA, Sigma) treatment for 45 minutes, followed by trypsin (2 mg/ml, Type II-S, Sigma) for 15 minutes at 37°C. The enzymatic reaction was stopped by washing the cells with DMEM containing 10% fetal bovine serum, and the remaining pieces of ganglia were gently triturated by using a fire-polished glass Pasteur pipette and passed through a 40-µm cell strainer. The suspension was then centrifuged at 800 rpm for 3 minutes, and the cell pellet was resuspended in fresh DMEM supplemented with 10% fetal bovine serum. The dissociated cells were placed on poly-D-lysine (0.1 mg/ml, Sigma)-treated glass coverslips contained within 4-well sterile tissue culture plates and kept in 5% CO_2_ incubator at 37°C for 2 to 3 hours before recording.

Whole-cell patch clamp recordings from acutely dissociated DRG neurons were performed using an EPC-10 amplifier and Patchmaster software (HEKA, Freiburg, Germany). Patch pipettes were pulled from borosilicate glass capillaries with a tip resistance of 5 to 8 MΩ when filled with internal solution containing the following (in mM):135 CsF, 10 NaCl, 10 HEPES, 5 EGTA and 2 Na_2_ATP. The pH was adjusted to 7.3 using CsOH. The external solution contained the following (in mM):30 NaCl, 20 TEA-Cl, 90 choline-Cl, 3 KCl, 1 CaCl_2_, 1 MgCl_2_, 10 HEPES, 10 glucose and 0.1 CdCl_2_. The pH was adjusted to 7.3 using Tris-base. Membrane currents were measured with pipette and membrane capacitance cancellation, filtered at 2 kHz and digitized at 10 kHz.

Under voltage-clamp recording mode, the cells were clamped at −70 mV, and series resistance was compensated to 70%–90%. The membrane capacitance was acquired from the amplifier by Patchmaster software for determining the size of cells and calculating the current density. All recordings were performed on small- and medium-diameter (20–35 µm) DRG neurons, which are known to express both TTX-S and TTX-R sodium currents [14], [15]. TTX-R currents were evoked from a holding potential of −120 mV to the test pulses ranging from −80 to +45 mV in increments of 5 mV at a frequency of 0.2 Hz in the presence of TTX (300 nM) to block all TTX-S channels. A Nav1.8-mediated sodium current was elicited with a voltage-clamp protocol used by Rush and Waxman [16] and Berta et al. [17] in the presence of 300 nM TTX. Test pulses ranging from −80 to +40 mV in increments of 5 mV at a frequency of 0.2 Hz, were preceded by a 500-ms pre-pulse step at −40 mV, to inactivate TTX-R Nav1.9 mediated current. The maximal peak current at various voltages was used for current density analysis. The normalized activation curves (I/I_0_) were fitted using the following Boltzmann distribution equation: I/I_0_ = 1-1/{1+exp[(V_1/2_-V_m_)/k]}, where I_0_ is the maximal peak current at various test voltages, V_m_ is the test potential, V_1/2_ is the membrane potential at half-maximal I, and k is the slope factor. The voltage-dependent steady-state inactivation was estimated by measuring the peak current amplitude elicited by a 100-ms test pulse to −10 mV after a 500-ms pre-pulse to potential over the range of −80 to +10 mV with a 20-s inter-pulse period. The normalized inactivation curves (I/I_0_) were fitted using the following Boltzmann distribution equation: I/I_0_ = 1/{1+exp[(V_1/2_-V_m_)/k]}, where I_0_ is the peak sodium current at tested pulse measured from the most negative preconditioning pulse potential, V_m_ is the preconditioning pulse potential, V_1/2_ is the membrane potential at half-maximal I, and k is the slope factor. Data analysis and fitting were performed using Origin software 8.5 (OriginLab Corporation, Northampton, MA).

### Western blot

Rats were deeply anesthetized with 10% chloral hydrate (0.3 g/kg, i.p.), and then the L4 and L5 DRGs were removed and immediately homogenized according to the protocol described in our previous report [18] for total protein extraction and assay. For cytosol or membrane protein extraction and separation, DRGs tissues were dissected and lysed by homogenization with Nucl-Cyto-Mem preparation kit (Applygen, China) according to the manufacturer's instructions, and then assayed using methods described by Black et al. [19]. Ten micrograms of total protein was mixed with 4× loading buffer and then subjected to SDS-PAGE. After blocking with 5% nonfat milk in Tris-buffered saline and Tween (TBST, 20 mM Tris-HCl (pH 7.5), 150 mM NaCl and 0.05% Tween-20) for 60 minutes at room temperature, the PVDF membranes were incubated with the following primary antibodies at 4°C overnight: rabbit anti-rat Nav1.8 antibody (1∶1000, Alomone Labs), mouse anti-β-actin (1∶3000, Santa Cruz Biotechnology), or mouse anti-rat transferrin receptor (TfR) antibody (1∶2000, Invitrogen). The blots were washed in TBST and then incubated in horseradish peroxidase–conjugated goat anti-rabbit/mouse IgG secondary antibody (1∶2000, Santa Cruz Biotechnology). Protein bands were visualized using an enhanced chemiluminescence detection kit (Pierce) followed by autoradiography using Hyperfilm MP (Santa Cruz Biotechnology). Blots were scanned with a cannon scanner (Cannon, Inc., Japan), and the band densities were detected with Quantity one software (Bio-Rad, USA). Data from five rats were used for statistical analysis.

### Implantation of intrathecal catheter and injection of drugs

For pharmacological blockade of the Nav1.8 channels, catheters were implanted at the same time with MRMT-1 tumor cells or PBS inoculation. Under chloral hydrate (0.3 g/kg, i.p.) anesthesia, implantation of intrathecal cannula was performed as described in our previous study [20]. Briefly, a PE-10 polyethylene catheter was implanted between the L5 and L6 vertebrae to reach the lumber enlargement of the spinal cord. The outer part of the catheter was plugged and fixed onto the skin on closure of the wound. All surgical procedures were performed under sterile conditions. Rats showing neurological deficits after the catheter implantation were excluded.

To examine effects of pharmacological blockade of Nav1.8 with a selective antagonist A-803467 (TOCRIS, UK) on bone cancer rats, A-803467 (50, 100 and 150 nmol) or its vehicle (1% DMSO) was intrathecally delivered to MRMT-1 rats on day 14 after the inoculation of tumor cells when pain hypersensitivity appears. As a control, high dose of A-803467 (150 nmol) or its vehicle (1% DMSO) was also intrathecally administrated to PBS inoculated sham animals on day 14 after PBS inoculation. Drug or vehicle was intrathecally injected via the implanted catheter in a 10-µl volume of solution followed by 10 µl of normal saline (NS) for flushing. Each injection lasted at least 5 min. After an injection, the needle remained in situ for 2 min before being withdrawn. Both pain behaviors and locomotor function of animals were evaluated at 15, 30, 60, 90 and 120 min after drug application.

### Assessment of mechanical allodynia

Mechanical allodynia, as a behavioral sign of bone cancer pain, was assessed by measuring 50% paw withdrawal threshold (PWT) as described in our previous reports [3], [21]. The 50% PWT in response to a series of von Frey filaments (Stoelting, Wood Dale, IL, USA) was determined by the Up and Down method [22]. Rats were placed on a metal mesh floor covered with an inverted clear plastic cage (18×8×8 cm) and allowed a 20-min period for habituation. Eight von Frey filaments with approximately equal logarithmic incremental (0.224) bending forces were chosen (0.41, 0.70, 1.20, 2.00, 3.63, 5.50, 8.50, and 15.10 g). Each trial started with a von Frey force of 2.00 g delivered perpendicularly to the plantar surface of the left hindpaw for about 2–3 seconds. An abrupt withdrawal of the foot during stimulation or immediately after the removal of the hair was recorded as a positive response. Whenever there was a positive or negative response, the next weaker or stronger filament was applied, respectively. This procedure was done until six stimuli after the first change in response had been observed. The 50% PWT was calculated using the following formula: 50% PWT = 10^(X^_f_^+kδ)^, where X_f_ is the value of the final von Frey filament used (in log units), k is a value measured from the pattern of positive/negative responses, and δ = 0.224, which is the average interval (in log units) between the von Frey filaments [23]. If an animal responded to the lowest von Frey filament, a value of 0.25 g was assigned. If an animal did not respond to the highest von Frey filament, the value was recorded as 15.0 g. Testing sessions were performed at 0, 15, 30, 60, 90 and 120 min after drug injection.

### Assessment of thermal hyperalgesia

Thermal hyperalgesia of the hind paw was tested as described by our previous report [20]. Rats were allowed to acclimate for a minimum of 30 min within acrylic enclosures on a clear glass plate maintained at 30°C. A radiant heat source was focused onto the plantar surface of the hind paw. Measurements of paw withdrawal latency (PWL) were taken by a timer that was started by the activation of the heat source and stopped when withdrawal of the paw was detected with a photodetector. A maximal cut-off time of 30 s was used to prevent unnecessary tissue damage. Three measurements of PWL were taken for each hind paw and were averaged as the result of each test session. The hind paw was tested alternately with greater than 5 min intervals between consecutive tests.

### Assessment of locomotor function

Inclined-plate test was performed for the assessment of locomotor function to rats received A-803467 (150 nmol). The rat was placed crosswise to the long axis of an inclined plate. The initial angle of the inclined plate was 50°. The angle was then adjusted in 5-degree increments. The maximum angle of the plate on which the rat maintained its body position for 5 s without falling was determined according to the method reported previously [24].

### Statistical analysis

Statistical analyses were performed with GraphPad Prism 5.0 package (GraphPad Software, Inc., La Jolla, CA). All data were expressed as the mean ±SEM. One-way analysis of variance (ANOVA) followed by Dunnett's multiple comparison test or two-way ANOVA followed by the Bonferroni post-hoc test was used for multiple comparisons. Differences with P<0.05 were considered statistically significant.

## Results

### Increase in TTX-R sodium current in DRG neurons of rats with bone cancer pain

**Figure 1:**
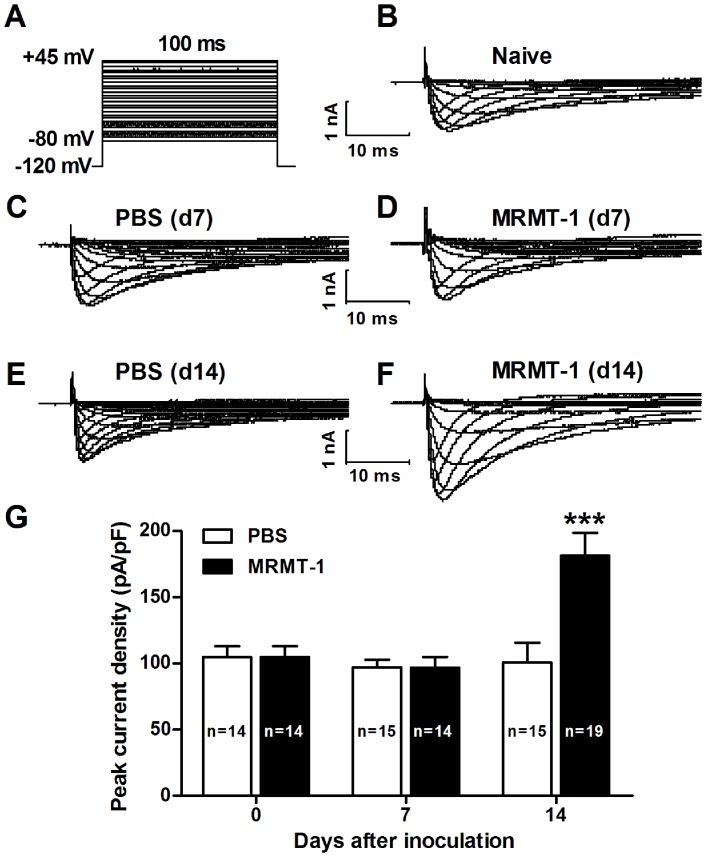
TTX-R sodium currents recorded on acutely isolated DRG neurons in naïve, PBS-treated and MRMT-1-treated rats. (A): Voltage protocol used for recording TTX-R sodium current with 300 nM TTX in the bath solution. (B to F): Representative traces of TTX-R sodium current in naïve (B), PBS-treated (C and E) and MRMT-1-treated (D and F) rats, recorded on DRG neurons at 7 (C and D) and 14 (E and F) days after the inoculation of tumor cells or PBS. (G): Statistical analysis of the peak current density. Note that the density of TTX-R sodium current is significantly increased in the DRG neurons of bone cancer rats. ^***^ *P*<0.001, compared to PBS group, two-way ANOVA, n = 19 (MRMT-1) and 15 (PBS).

To explore whether TTX-R sodium channels contribute to the development of cancer-induced bone pain, we first measured TTX-R sodium currents recorded on acutely isolated DRG neurons in a rat model of bone cancer pain, by adding TTX (300 nM) to the bath solution to block all TTX-sensitive channels [25]. The voltage protocol we used is shown in Fig. 1A. We found that the density of TTX-R sodium current increased gradually following the inoculation of tumor cells in a time-dependent manner. On day 7 after inoculation, no significant difference was observed on the density of TTX-R sodium current between neurons from rats treated with MRMT-1 tumor cells (96.67±8.2 pA/pF, n = 14) and those from rats treated with PBS (97.08±5.8 pA/pF, n = 15) (P>0.05, two-way ANOVA, Figs. 1C, D and G), which is consistent with our previous behavioral findings showing there was no mechanical or thermal pain hypersensitivity at that time [3]. However, on day 14 after inoculation, the density of TTX-R sodium current increased significantly in MRMT-1-treated rats (181.34±17.2 pA/pF, n = 19) compared to PBS- treated rats (100.83±14.7 pA/pF, n = 15) (P<0.001, two-way ANOVA, Figs. 1E to G). Representative traces of TTX-R sodium current in naïve, PBS-treated and MRMT-1-treated rats, recorded on DRG neurons at 7 and 14 days after the inoculation of tumor cells or PBS are shown in Figs. 1B to F.

### Increase in Nav1.8 sodium current in DRG neurons of rats with bone cancer pain

**Figure 2:**
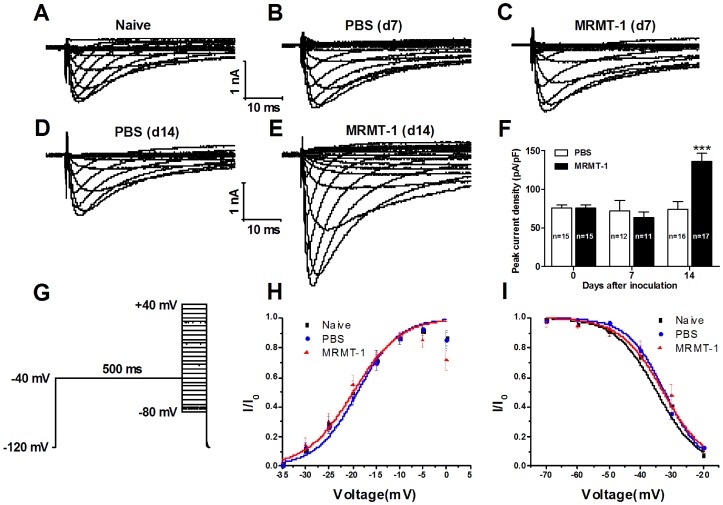
Nav1.8-mediated sodium current recorded on acutely isolated DRG neurons in naïve, PBS-treated and MRMT-1-treated rats. (A to E): Representative traces of Nav1.8-mediated sodium current in naïve (A), PBS- (B and D) and MRMT-1-treated (C and E) rats, recorded on DRG neurons at 7 (B and C) and 14 (D and E) days after the inoculation of tumor cells or PBS. (F): Statistical analysis of the peak current density. Note that the density of Nav1.8-mediated sodium current is significantly increased in the DRG neurons of bone cancer rats. ^***^ *P*<0.001, compared to PBS group, two-way ANOVA, n = 17 (MRMT-1) and 16 (PBS). (G): Voltage protocol used for recording Nav1.8-mediated sodium currents with 300 nM TTX in the bath solution. A 500-ms pre-pulse at −40 mV was used to inactivate Nav1.9 sodium channels. (H and I): Activation and inactivation curves of Nav1.8-mediated sodium current in naïve, PBS-treated and MRMT-1-treated rats at 14 days after the inoculation of tumor cells or PBS. Note that no significant alteration is observed either on the activation (H) or on the inactivation (I) curves among the three groups. Activation curves were fitted using the same protocol used for the recording of Nav1.8 sodium current. Note that the curves in Naïve and PBS groups overlap almost completely. The inactivation curves were fitted using the protocol described in the methods.

To isolate Nav1.8 sodium current from total TTX-R sodium current, we used another voltage protocol with a 500-ms pre-pulse at −40 mV to inactivate the Nav1.9 channel as described in previous report [17] (Fig. 2G). The results showed that the density of Nav1.8-mediated sodium current 14 days after inoculation increased significantly in MRMT-1-treated rats (136.11±10.4 pA/pF, n = 17) compared to PBS-treated rats (74.09±10.0 pA/pF, n = 16) (P<0.001, two-way ANOVA, Figs. 2D to F). Similar to the density of the TTX-R sodium current, the Nav1.8-mediated current density remained unchanged on day 7 after the inoculation of tumor cells (P>0.05, vs. PBS, two-way ANOVA, n = 11 MRMT-1 and 12 PBS, Figs. 2B, C and F). To investigate whether the intrinsic properties of Nav1.8 sodium channel were changed after the inoculation of tumor cells, we examined both the activation and inactivation curves of Nav1.8-mediated sodium currents in naïve, PBS-treated and MRMT-1-treated rats. As shown in Figs. 2H and I, no significant alteration was observed on either the activation (Fig. 2H) or the inactivation (Fig. 2I) curves among the three groups, indicating that the intrinsic properties of Nav1.8 sodium channel remained unchanged after the inoculation of tumor cells. Representative traces of Nav1.8-mediated sodium current in naïve, PBS- and MRMT-1-treated rats, recorded on DRG neurons at 7 and 14 days after the inoculation of tumor cells or PBS are shown in Figs. 2A to E.

### Increase in membrane expression of Nav1.8 protein in the DRG of rats with bone cancer pain

**Figure 3:**
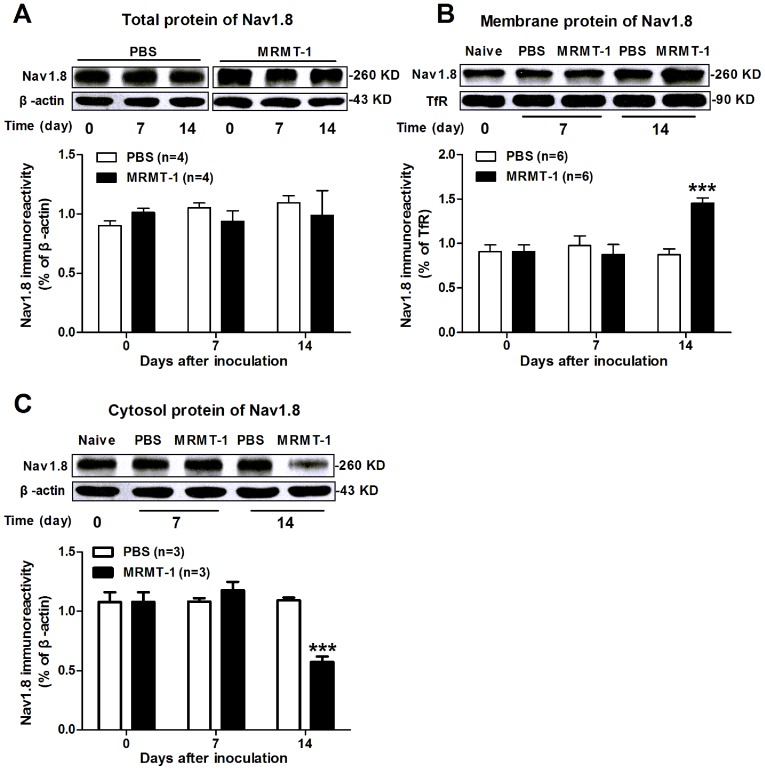
Expression of Nav1.8 protein in the DRG in naïve, PBS-treated and MRMT-1-treated rats. (A): Total Nav1.8 protein. Upper: representative of Western blot bands; Lower: statistical analysis of Nav1.8 total protein expression. No significant difference is observed on the expression of Nav1.8 total protein among the three groups. *P*>0.05, two-way ANOVA, n = 4/group. (B): Nav1.8 membrane protein. Upper: representative of Western blot bands. Transferrin receptor (TfR) is used as internal control. Lower: statistical analysis of Nav1.8 membrane protein expression. Note that the expression of Nav1.8 on the cell membrane is increased significantly in the DRG neurons of MRMT-1-treated rats compared to PBS-treated rats. ^***^ *P*<0.001, two-way ANOVA, n = 6/group. (C): Nav1.8 protein in the cytosol. Note that the expression of Nav1.8 in the cytosol is significantly decreased in the DRG neurons of MRMT-1-treated rats compared to PBS-treated rats. ^***^ *P*<0.001, two-way ANOVA, n = 3/group.

Furthermore, we examined expression of Nav1.8 protein in the DRG of rats with cancer-induced bone pain, in particular, the channels on the cell membrane, which represent functional channels. Using Western blot assay, we found that the expression of Nav1.8 total protein in ipsilateral L4 and L5 DRGs remained unchanged from day 7 to day 14 in MRMT-1-treated rats compared to PBS-treated rats (P>0.05, two-way ANOVA, n = 4/group, Fig. 3A). This was unexpected, as electrophysiology experiments revealed an increased current density of Nav1.8 in MRMT-1-treated rats (see Fig. 2). We speculate that such an increase of Nav1.8 sodium current is probably due to increased expression of Nav1.8 on the cell membrane because the intrinsic properties of Nav1.8 sodium channel remained unchanged after the inoculation of tumor cells (see Figs. 2H and I). To clarify this hypothesis, we further detected the membrane expression of Nav1.8 using a high-speed centrifugation method to isolate the membrane fraction from DRG cells as described in previous study [19]. As expected, 14 days after the inoculation of tumor cells, the band density of Nav1.8 protein expressed on the cell membrane increased markedly in MRMT-1-treated rats (1.45±0.06, n = 6) compared to PBS-treated rats (0.87±0.07, n = 6) (P<0.001, two-way ANOVA, Fig. 3B). Meanwhile, the band density of Nav1.8 protein located in the cytosol decreased markedly in MRMT-1-treated rats (0.57±0.04, n = 3) compared to PBS-treated rats (1.09±0.02, n = 3) (P<0.001, two-way ANOVA, Fig. 3C).

### A-803467 alleviates the mechanical allodynia and thermal hyperalgesia of bone cancer rats

**Figure 4:**
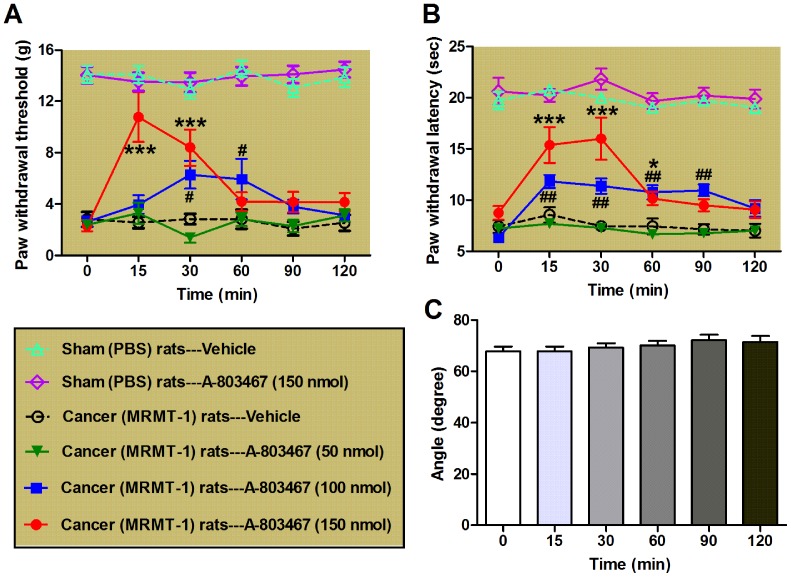
Effects of A-803467, a selective blocker of Nav1.8, on bone cancer-induced mechanical allodynia and thermal hyperalgesia in rats. (A, B): Effects of A-803467 (at 50, 100 and 150 nmol) on mechanical (A) and thermal (B) pain behaviors in rats evaluated on day 14 after the inoculation of MRMT-1 tumor cells or PBS. Note that A-803467 remarkably restores the tumor cells-induced reduction of PWT and PWL in cancer model rats but has no significant effect on the PWL and PWT in PBS inoculated sham rats. ^*/#^ *P*<0.05, ^**/##^ *P*<0.01, ^***^ *P*<0.001, compared to vehicle/cancer (MRMT-1) rats group, two-way ANOVA, n = 6–9/group. (C): Inclined-plate test. Note that intrathecal administration of A-803467 at the maximal dose of 150 nmol has no significant effect on the locomotor function of rats. *P*>0.05, one-way ANOVA, n = 7/group.

Finally, we investigated whether A-803467, a selective blocker of Nav1.8 [26] could alleviate the cancer-induced bone pain in rats. We tested PWT and PWL on day 14 after the inoculation of tumor cells or PBS as the base line. Then, A-803467 or vehicle DMSO was intrathecally delivered to MRMT-1 inoculated cancer pain rats as well as PBS inoculated sham rats, and both the PWT and PWL were measured at 15, 30, 60, 90 and 120 min after drug application. The dosage and injection root of A-803467 was according to a previous report showing that intrathecal administration of A-803467 (50–150 nmol) reduces both evoked and spontaneous discharges of wide dynamic range (WDR) neurons in the spinal dorsal horn [27]. As shown in Fig. 4, intrathecal administration of A-803467 (at both 100 and 150 nmol) could prominently restore the bone cancer-induced decrease in both PWT (P<0.05 to 0.001, vs. vehicle/cancer rats group, two-way ANOVA, n = 7–8/group, Fig. 4A) and PWL (P<0.05 to 0.001, vs. vehicle/cancer rats group, two-way ANOVA, n = 6–9/group, Fig. 4B) of cancer rats. By contrast, even a high dose of A-803467 (150 nmol) had no significant effect on the PWL and PWT of PBS inoculated sham rats (P>0.05, vs. vehicle/sham rats group, two-way ANOVA, n = 6/group, Figs. 4A and B). To exclude any possible motor dysfunction induced by A-803467, we also examined effect of A-803467 (at a maximal dose of 150 nmol) on the locomotor function of rats using inclined-plate test. As our expectation, no significant motor dysfunction was observed at any time point after drug injection (p>0.05, one-way ANOVA, n = 7/group, Fig. 4C). These data suggest that the inhibition of Nav1.8 by A-803467 may rescue the tumor-induced mechanical allodynia and thermal hyperalgesia in bone cancer rats.

## Discussion

### Functional upregulation of Nav1.8 sodium channels in DRG neurons of rats with bone cancer pain

Consistent with previous findings that the expression of Nav1.8 mRNA and protein are increased within lumbar 4–5 DRG in an animal model of bone cancer pain [10], our present study provide additional direct evidence demonstrating a functional upregulation of voltage-gated sodium channel subtype Nav1.8 on the membrane of DRG neurons in a rat cancer pain model, which is proved to be required for the development of cancer-induced bone pain.

Biophysical and pharmacological studies have identified that four peripheral-specific sodium channels including the TTX-S channels NaV1.3 and NaV1.7 and the TTX-R channels NaV1.8 and NaV1.9 are preferentially expressed on primary sensory DRG neurons and play important roles in the pathophysiology of different pain syndromes [18], [28], [29]. Among them, the Nav1.8 sodium channel has been shown to be necessary for many types of chronic inflammatory and neuropathic pain [26], [30], [31]. However, the role of Nav1.8 sodium channel in the pathogenesis of bone cancer pain is still unknown. Although Qiu et al. [10] have observed an increased expression of Nav1.8 within DRG in a rat model of bone cancer pain, they did not definitely draw a conclusion whether or not the increased Nav1.8 sodium channel contributes to the cancer-induced bone pain. In another study, Miao and colleagues [32] have reported a significant downregulation of Nav1.8 expression in the DRG in a rat cancer pain model. Nevertheless, using antisense oligodeoxynucleotides (ODNs) against Nav1.8, they found that knock-down of Nav1.8 expression in DRG neurons could alleviate established cancer pain behaviors in tumor-bearing rats, implying a possible role of Nav1.8 in the development and maintenance of bone cancer pain. Inconsistently, a recent study using conditional knock-out mice has shown that neither Nav1.7 or Nav1.8 is required for cancer-induced bone pain [33]. In our current study, we present electrophysiological and biochemical evidence showing that the density of Nav1.8-mediated sodium current is significantly increased in the DRG neurons of rats with bone cancer pain and that this increase is probably due to an increased expression of functional Nav1.8 sodium channels on the cell membrane instead of changes in intrinsic properties or total protein expression of the channels, because that, along with the enhanced density of both TTX-R- and Nav1.8-mediated sodium currents, the expression of membrane protein rather than the total Nav1.8 protein is significantly increased, whereas the Nav1.8 protein level in the cytosol is markedly decreased after the inoculation of tumor cells to rats. Together with the electrophysiological findings showing that the intrinsic properties, such as voltage-dependent activation and inactivation of Nav1.8 sodium channels, remained unchanged after the inoculation of tumor cells, the increased expression of Nav1.8 on the cell membrane provides a possible explanation for the enhanced density of Nav1.8- mediated sodium current in the DRG neurons of MRMT-1-treated rats, which actually represents the functional upregulation of Nav1.8 sodium channels in the DRG neurons of bone cancer rats. Certainly, we can not exclude other possible explanations such as changes in channel conductance and open probability in our present study. To address these questions, single channel recordings should be done to determine whether or not the properties of the channel are changed in the future study.

It is worthy of note that the increase in membrane expression of Nav1.8 and the enhancement of Nav1.8-mediated sodium currents density are highly coincident at the time point, that is, both of them are enhanced apparently on day 14 but not day 7 after the inoculation of tumor cells in rats. These findings are in agreement with previous reports that the upregulation of Nav1.8 mRNA and protein levels within DRG appears at the same time in tumor cells inoculated rats [10]. In fact, a previous study in our lab has shown that the cancer-induced pain hypersensitivity actually emerges on day 14 but not day 7 after the inoculation of cancer cells in rats [3]. As to Nav1.8 protein expression, another interesting finding in our present study is that the membrane protein but not the total protein of Nav1.8 is increased whereas the Nav1.8 protein level in the cytosol is decreased in cancer rat DRGs. One possible explanation is the trafficking of the channels from the cytosol to the membrane of the cell, which probably underlies the enhanced density of Nav1.8-mediated sodium currents in DRG neurons of cancer pain model rats. Truthfully, we can not give an adequately explanation for the discrepancy of our observations on Nav1.8 expression compared to previous report of a significant downregulation in a rat cancer pain model [32]. We speculate that differences in animal species (Sprague-Dawley vs. Wistar rats), the inoculated tumor cells (MRMT-1 vs. Walker 256 tumor cells), and the observed time points (day 7 to day 14 vs. day 16 to day 19 after surgery) between our two groups probably result in the discrepancy. Surprisingly, their findings that knock-down of Nav1.8 expression in DRG neurons alleviates established cancer pain behaviors in tumor-bearing rats [32], supporting the contribution of Nav1.8 in the development of bone cancer pain.

### Contribution of upregulated Nav1.8 sodium channels in DRG neurons to the development of bone cancer pain

Nav1.8 has been shown to contribute most of the sodium current underlying the upstroke of action potentials [8], [9], [29], [34], and thus its modulation can significantly influence neuronal excitability. The distinguishing features of the depolarized activation and inactivation, and rapid repriming of Nav1.8 sodium channel enable it has the ability for regulating the repetitive firing of the neurons that express it [34], [35]. Therefore, a functional upregulation of Nav1.8 sodium channels on DRG neurons will probably account for the enhanced excitability of these neurons in cancer state [3], thereby playing a critical role in the pathogenesis of bone cancer pain. Actually, we found that blockade of Nav1.8 sodium channels on DRG neurons by intrathecal administration of A-803467, a selective blocker of this channel [26], did in fact alleviate the tumor-induced mechanical allodynia and thermal hyperalgesia in a dose-dependent manner in bone cancer rats. However, as a control, even a high dose of A-803467 (150 nmol) had no significant effect on the PWL and PWT of PBS inoculated sham rats. These findings are consistent with previous report showing that knock-down of Nav1.8 expression in DRG neurons alleviates established cancer pain behaviors in tumor-bearing rats [32], suggesting it is the enhancement of Nav1.8 sodium channels on DRG neurons responsible for the cancer-induced pain hypersensitivity. In support of this notion, accumulative evidence has shown that inhibition of Nav1.8 sodium channels with A-803467, can effectively block both evoked and spontaneous neuronal action potentials in vitro [26] and attenuate the firing of spinal wide dynamic range (WDR) neurons in vivo [27], and therefore alleviate mechanical allodynia and thermal hyperalgesia in a variety of neuropathic and inflammatory pain models [26], [30]. In addition, antisense studies have also shown an important role of Nav1.8 channels in animal models of inflammatory pain [31], [36] and neuropathic pain [31], [37], whilst using small interfering RNA (siRNA) to specifically knock down of Nav1.8 expression in DRG neurons reverses mechanical allodynia in neuropathic rats [38]. However, ablation of Nav1.8 in DRG neurons does not diminish the development of cancer-induced bone pain in a mouse model [33]. Given the facts that knock-down of Nav1.8 expression [32] or selective blockade of Nav1.8 channels inhibits cancer-induced pain behaviors (see Fig. 4), it is possible that genetic compensatory mechanisms mask some role of Nav1.8 in the pathogenesis of bone cancer pain in the knockout mouse. Indeed, an upregulation in the levels of TTX-S sodium channel Nav1.7 [7] and a more hyperpolarized voltage-dependence of activation for TTX-S channels [39] have been reported in Nav1.8-null DRG neurons. An augmentation of TTX-S sodium density or a change in their gating properties is also proposed underlie the normal excitability of nerve terminals in Nav1.8-null mice [40].

In conclusion, our present results provide strong evidence showing that functional upregulation of Nav1.8 sodium channels on the cell membrane of DRG neurons contributes to the development of cancer-induced bone pain in rats.
